# Medical and Physical Intelligence

**Published:** 1820-07

**Authors:** 


### Medical and Physical Intelligence.

MEDICAL STATISTICS, *relative to Paris; extracted from the Bills of Mortality drawn up by the Twelve Municipalities, for the Year* 1819.—The number of deaths was 22,137: in addition to which are to be reckoned 376 by suicide; 250 of which were of males, and 126 of females. Of the 22,137 deaths, 14,827 occurred in private residences, 7,321 being men, and 7,506 women; and 7,310 in hospitals, of which 3,620 were males, and 3,690 females. The number of deaths from small-pox was 169.

The 22,137 deaths above mentioned comprises 245 of bodies deposited at *La Morgue*,* 193 of which were males, and 52 females.

The principal causes of mortality were as follows:

| | Males. | Females. | Total. |
|---|---|---|---|
| Fevers, putrid or adynamic | 363 | 449 | 812 |
| Mucous | 68 | 125 | 193 |
| Malignant | 370 | 396 | 768 |
| Indeterminate | 274 | 326 | 600 |
| Phlegmasiæ, cutaneous | 445 | 379 | 824 |
| of the mucous membranes | 1,336 | 1,607 | 2,943 |
| of the serous membranes | 220 | 217 | 437 |
| of the cellular tissue and parenchymatous organs | 1,478 | 1,876 | 3,354 |
| Comatous affections | 501 | 431 | 932 |
| Spasmodic affections | 1,038 | 905 | 1,943 |
| Local nervous affections | 583 | 514 | 1,097 |
| Organic lesions, general | 1,917 | 1,903 | 3,820 |
| partial | 774 | 901 | 1,675 |
| Gangrenous inflammations | 55 | 110 | 165 |
| Puerperal women | 0 | 179 | 179 |
| Still-born children | 672 | 526 | 1,198 |

* A place at Paris, where the dead bodies of unknown persons are exposed to the public, with the clothes usually worn by them, in order that they may be recognized by their relations. A great proportion of the bodies thus exposed have, ordinarily, been found drowned, and are probably those of suicides.—Edit.

The births amounted to 23,263; 11,871 of which were of the male sex, and 11,392 of the female.

The periods at which death took place after birth, were as follows:

| | Males. | Females. | Total. |
|---|---|---|---|
| From the time of birth to 3 months | 2,250 | 1,803 | 4,053 |
| 3 to 6 months .... | 204 | 163 | 367 |
| 6 months to 1 year | 421 | 427 | 848 |
| 1 year to 2 years | 711 | 717 | 1,428 |
| 2 years to 3 — | 428 | 400 | 828 |
| 3 — 4 — | 284 | 274 | 558 |
| 4 — 5 — | 185 | 171 | 356 |
| 5 — 6 — | 132 | 136 | 268 |
| 6 — 7 — | 106 | 119 | 225 |
| 7 — 8 — | 59 | 87 | 146 |
| 8 — 9 — | 46 | 56 | 102 |
| 9 — 10 — | 64 | 45 | 109 |
| 10 — 15 — | 205 | 228 | 433 |
| 15 — 20 — | 372 | 360 | 732 |
| 20 — 25 — | 353 | 544 | 897 |
| 25 — 30 — | 303 | 472 | 775 |
| 30 — 35 — | 285 | 347 | 632 |
| 35 — 40 — | 286 | 396 | 682 |
| 40 — 45 — | 268 | 410 | 678 |
| 45 — 50 — | 330 | 460 | 790 |
| 50 — 55 — | 459 | 436 | 922 |
| 55 — 60 — | 456 | 452 | 908 |
| 60 — 65 — | 555 | 554 | 1,109 |
| 65 — 70 — | 600 | 633 | 1,233 |
| 70 — 75 — | 542 | 546 | 1,088 |
| 75 — 80 — | 384 | 522 | 906 |
| 80 — 85 — | 214 | 326 | 540 |
| 85 — 90 — | 85 | 150 | 235 |
| 90 — 95 — | 14 | 23 | 37 |
| 95 — 100 — | 0 | 6 | 6 |

---

In our review of Dr. Cooke's work on *Apoplexy*, in the last Number of this Journal, we mentioned that the author intended to produce a treatise of a similar character on *Palsy* and *Epilepsy*. Numerous medical practitioners must have made many original observations relative to the latter diseases, which they may not, individually, consider sufficient to form the subject of a memoir, and yet are more or less valuable in a history of them. Dr. Cooke has expressed a desire to receive accounts of such observations from the profession generally; as a collection of all the existing knowledge that has never been recorded in literature, must prove a very important addition to that derived from the latter source. It is but reasonable to expect that the older members of the profession should endeavour to aid, in the way just mentioned, Dr. Cooke's exertions to effect the object he aims at in the treatise alluded to. These works must save students an immensity of time and labour in their researches; and they will be not much less valuable to practitioners, by presenting to them, in a methodic form, all the most important information that has been acquired respecting the nature and treatment of several of the most serious maladies. Every man, we think, must feel a desire to be permitted to contribute to such a work.

---

Mr. Pagenstecher, of Berne, has discovered a very sensible reactive for the detection of copper in solution. He says, "If we drop into a newly-prepared tincture of guaiacum-wood a concentrated solution of salt of copper, the mixture instantly assumes a blue colour. This effect does not take place when the solution is very weak, and does not contain above half a grain of the salt in an ounce of water: however, by the addition of a few drops of prussic acid, the blue colour is then immediately developed with the utmost purity and intensity. This colour is not permanent; it soon passes to a green, and at length totally disappears. In want of prussic acid, distilled laurel-water, or that of plum or black-cherry kernels, may be employed. This reactive succeeds when the proportion of salt to the liquid is not more than as 1 to 45,000: in this proportion, no other test, whether the prussiates of potash, of soda, or of ammonia, will develop the least indication of the presence of copper."

In the use of the tincture of guaiacum as a test for copper, it must be remembered that the resin of wood assumes a blue colour on admixture with other bodies.—*Annales Générales des Sciences Physiques.*

---

The Professors at the Veterinary School at Alfort have been repeating some experiments, made long since in England, on the inoculation of the *glanders* of horses. Some pieces of the mucous membrane of the nostrils were placed under the skin of the chest of a strong horse: a gangrenous affection of the part, similar to what is called white carbuncle, soon appeared, and the animal died on the fourth day after the inoculation.

The following experiments on the division of the *par vagum* in horses, are somewhat interesting. When division of the two nerves is effected about the middle of the neck, and especially if an opening has been previously made in the trachea, the animal, instead of dying in a few hours, as is the case with some others, lives for five or six days: he continues to eat and drink, the stomach and œsophagus *becoming filled*, and the inferior laryngeal nerves being paralyzed, the food is soon observed to pass out of the opening in the trachea; the passage of these foreign matters soon produces gangrene of the parts, all the symptoms of carbuncular typhus appear, and the animal soon dies.

When only one of the par vagum is wholly divided, and the other two-thirds cut through, and tracheotomy is not performed, the animal breathes without making any noise when he walks; but, when he is made to trot, respiration becomes sonorous, and like that of horses which are callen *broken-winded.*

An interesting experiment was made at the same School on a broken-winded horse, in whom the affection was so bad, that it could not be made to trot without danger of suffocation. The operation of tracheotomy was performed, and a large tube introduced into the opening. For fifteen months since, the animal has breathed only through this tube: it is in very good condition, works well, and has often gone twelve or fifteen leagues in a day, drawing a cabriolet with two or three persons.

We learn from the same source, that M. Barthelemy has cured, in a very short time, many horses with the *farcy*, where the affection was general, by the use of kermes mineral (sub-hydro-sulphate of antimony), given in large doses.

---

*Prize Question.*—The Society of Sciences of Copenhagen have proposed the following:

"Quibus naturæ legibus regitur primaria evolutio corporum animalium, et formam sive regularum, normalem, sive abnormem adsciscant?"

The author of the best answer to this question will receive a gold medal of the value of fifty ducats. The memoirs should be addressed, with the usual forms, before the end of December 1820, to the Secretary of the Society, Professor Oersted, at Copenhagen.

### REPORT OF DISEASES.

UNTIL the latter end of the month, pulmonary affections have been the prevalent forms of disease, and the mortality from them, especially in children, has far exceeded the ordinary rate at this season of the year. We have had many opportunities for examination after death, principally in the practice of a Dispensary, and have found one form of disease of the lungs to be by far of the most common occurrence. The lower lobes of the lungs, most frequently on both sides, are found after death very much engorged with blood, partially hepatized; and, on being cut into by the knife, a large quantity of sanguineous frothy fluid has sprung out at every incision: the pleura has been less commonly inflamed, and its two surfaces adherent; the mucous membrane of the bronchiæ corresponding to the state of the lungs above described, has been of a bright-red colour, coated with thick mucus, and this redness and vascularity have extended upwards, sometimes to the extremity of the larynx, at others only to the upper part of the trachea. These cases have, in a considerable proportion, been very rebellious to the most active means that could be suggested for their relief. In a few cases in adults, where we have suspected such a state of the lungs to exist, and when bleeding, blisters, antimony, &c. had been employed vigorously in the first stages, and when the patient has been apparently dying, with a full, soft, pulse, conveying an idea, by the touch, that the artery had but little elasticity, the subcarbonate of ammonia, with squills and ipecacuanha, has produced a rapid change for the better in the condition of the patient. This plan has been pursued about two or three days, and then followed by a mixture of squills, ipecacuanha, and spirit of nitrous ether, and some ordinary auxiliary measures, which have led the disorder to a favourable termination. The ammonia is a very powerful and efficacious agent in such cases; but much caution is required in distinguishing the cases to which it is applicable, and it is never appropriate for the earlier stages of the disease.

The chronic rheumatism of the lower class of people very frequently gives way to the vinum colchici; and we have not found any important ill effects from this remedy, in a very extensive series of cases. We have not found it so generally efficacious in the same kind of affection as it occurs in the upper class of society, though it is here often successful.

Scarlet fever has begun to appear in the more confined parts of the town, and is, in a considerable proportion of instances, attended with unusual pulmonary affection. We have not yet seen a fatal case of it.

### *Note from Dr. Burrows respecting the Review of his Work, in the last Number of this Journal.*

SIR,

IN the critique on my "Inquiry into certain Errors relating to Insanity, which appeared in the last Number of the Medical and Physical Journal, in summing up his opinion, the reviewer observes, that it is "an inferior specimen of medical literature, as far as literature is concerned."

Permit me to remark, that I have, in pages 15, 192, and 219, sufficiently intimated, that I do not offer it as a contribution to medical literature; but have, as the reviewer himself has quoted, "adopted a familiar form, avoiding all technicality and *medical reasoning*." Consequently, it is scarcely fair to condemn it for not being that which it was never professed to be.

Far be it from me to arraign the judgment of any reviewer; but, as matter of fact, perhaps you will allow me to add, that I have no recollection of having used a word in any writing I ever published, that originated with me. Whether the words objected to are appropriate or otherwise, depends probably on taste; and I am willing to submit them to the decision of the public.

I am, Sir, your obedient servant,

G. M. BURROWS.

62, *Gower-street; June* 1820.

To the Editor of the Medical and Physical Journal.

---

### *Remarks on the foregoing Note, by the Reviewer.*

THE Reviewer must, in the first instance, express his grateful thanks to Dr. BURROWS, for pointing out with so much politeness the passage cited in his note from the critique on his work, in which the Reviewer expressed his sentiments in language that, it appears, will admit of inferences very different from those which he intended should be drawn from it. The Reviewer did not mean to state, that the work of Dr. Burrows is an inferior production in its *medical* character; but merely that, "as far as *literature* (that is, expressly, language and style), is concerned," it is not a good specimen of medical literature. The Reviewer thought that this sentiment was clearly expressed in the passage in question; especially since it followed a recommendation of the work to the perusal of those who devote their attention to the part of medical police on which it treats; and the remarks, that it contains "much interesting information," and "such as could not have been procured without considerable exertions:" expressions indicative of superior merit, as far as relates to its medical character.

In reply to the remark of Dr. Burrows, that "it is scarcely fair to condemn it for not being that which it was never professed to be," the Reviewer has only to say, that he did not consider the statement of the author, that, "as the subject is one in which the welfare of every individual is more or less involved," he had "preferred a familiar form, avoiding as much as possible all technical language and medical reasoning," should prevent the work being regarded as medical literature, and being subservient to criticism as such, in the point of view in which the writer of the critique in question had thus treated it. The propriety of his opinions in this respect, as well as on the language of the author in an abstract manner, are not here insisted on: the writer merely wishes to disclose the sentiments which prompted him to express the opinions in question.

With respect to the remarks on the new words: the Reviewer could not recollect having witnessed the use of some of them by any former writer, and three of them are not in the latest English dictionary (that of Todd), which professes to give all those in use: he therefore was led to attribute the original introduction of them to Dr. Burrows.

On re-perusing Dr. Burrows's suggestions respecting the existing legislative regulations in regard to lunatics, the Reviewer has been struck by his remarks on the laws relating to the superintendents of lunatic asylums, and the certificates of medical practitioners. At first, the Reviewer considered those laws as formed on the same spirit as those directing *ex-officio* informations; but it now appears to him that they are much more severe, since the line of conduct marking delinquency is not by any means discernible, with equal readiness, in both the instances above alluded to; and he acknowledges the importance of Dr. Burrows' arguments for the abolition of them. "Superintendents of licensed asylums," he states, "are exposed, under the present Act of Parliament, as they would have been under the rejected Bills, to be prosecuted for receiving a lunatic, even though they complied with every form which the Act imposes, to guard against improper objects being admitted into such houses. This surely is a manifest injustice: for, as the superintendent has no prior knowledge of the patient or his case, and receives him upon the report and authority of those whom the law entitles to decide and act, he ought not to be made responsible for the deeds of others."—"So likewise, medical gentlemen who sign a certificate of lunacy, founded on a deliberative examination of the patient, and in strict conformity with the letter and spirit of the law, ought not to be, as they now are, liable to actions for trespass, or indictments for a conspiracy; unless it be proved, by the previous conviction of the persons by whose order he states he acted, that there was some collusion or connivance. Even although honourably acquitted of all imputation by the verdict of a jury, yet, so jealous are the people of this country in all that relates to lunatics, that suspicion will often still attach; and to a certainty his defence will entail a very heavy expense on him."

### METEOROLOGICAL JOURNAL.

By Messrs. WILLIAM HARRIS and Co. 50, Holborn, London.

*From May 20 to June 19, inclusive.*

| Day of Month. | Moon. | Rain gauge | THERM. | | | BAROM. | | De Luc's HYGROM. Dry | Damp | | WIND. | | ATMOSPHERIC VARIATION. | | |
|---|---|---|---|---|---|---|---|---|---|---|---|---|---|---|---|
| May | | | | | | | | | | | | | | | |
| 20 | ☽ | | 60 | 64 | 51 | 30·15 | 30·30 | | 10 | 9 | WSW | WSW | Fine | Cloud. | Cloud. |
| 21 | | | 62 | 66 | 53 | 30·33 | 30·29 | | 8 | 8 | WSW | SW | Fine | | |
| 22 | | | 62 | 70 | 54 | 30·21 | 30·11 | | 9 | 10 | S | SE | Fine | | |
| 23 | | | 62 | 73 | 57 | 30·00 | 29·86 | | 11 | 9 | SSE | E | Fine | | |
| 24 | | | 63 | 71 | 51 | 29·80 | 29·80 | | 8 | 8 | SE | S | Fine | Fine | Cloud. |
| 25 | | ‘50 | 55 | 59 | 49 | 29·79 | 29·74 | | 9 | 9 | SW | SW | Rain | Sho'ry | Cloud. |
| 26 | | ‘03 | 61 | 62 | 52 | 29·84 | 29·76 | | 8 | 7 | W | WSW | Cloud. | Sho'ry | Cloud. |
| 27 | ○ | ‘47 | 53 | 55 | 52 | 29·67 | 29·65 | | 8 | 7 | WNW | WSW | Rain | Fine | Cloud. |
| 28 | | ‘03 | 58 | 62 | 50 | 29·60 | 29·50 | | 6 | 8 | W | W | Cloud. | Rain | Sho'ry |
| 29 | | ‘15 | 53 | 58 | 48 | 29·40 | 29·35 | | 6 | 5 | WNW | W | Cloud. | Rain | Fine |
| 30 | | ‘01 | 50 | 58 | 49 | 29·38 | 29·38 | | 4 | 5 | W | W | Fine | Rain | Sho'ry |
| 31 | | ‘02 | 54 | 58 | 48 | 29·40 | 29·46 | | 4 | 5 | W | W | Cloud. | Rain | Cloud. |
| June | | | | | | | | | | | | | | | |
| 1 | | ‘01 | 54 | 56 | 50 | 29·51 | 29·57 | | 45 | 44 | W | W | Fine | Rain | Fine |
| 2 | | ‘32 | 56 | 59 | 47 | 29·60 | 29·68 | | 44 | 45 | W | W | Cloud. | Rain | Rain |
| 3 | ☾ | ‘07 | 51 | 57 | 46 | 29·76 | 29·84 | | 45 | 48 | WNW | NW | Cloud. | Rain | Cloud. |
| 4 | | ‘01 | 50 | 52 | 46 | 29·91 | 29·91 | | 47 | 48 | WNW | WNW | Rain | Sho'ry | Cloud. |
| 5 | | | 52 | 60 | 50 | 30·00 | 29·91 | | 48 | 48 | WNW | WNW | Cloud. | Fine | Sho'ry |
| 6 | | ‘25 | 53 | 56 | 48 | 29·95 | 30·08 | | 48 | 47 | NNW | NE | Sho'ry | Cloud. | Fine |
| 7 | | | 54 | 60 | 48 | 30·07 | 29·97 | | 50 | 49 | WNW | N | Fine | Cloud. | Cloud. |
| 8 | | | 55 | 62 | 54 | 29·97 | 29·90 | | 49 | 51 | NNW | NW | Cloud. | Fine | Fine |
| 9 | | ‘06 | 56 | 60 | 47 | 29·81 | 29·73 | | 51 | 50 | NW | NW | Cloud. | Cloud. | Rain |
| 10 | ● | | 52 | 60 | 47 | 29·76 | 29·70 | | 50 | 51 | NNW | NW | Cloud. | Cloud. | Fine |
| 11 | | ‘02 | 53 | 57 | 50 | 29·59 | 29.61 | | 50 | 52 | W | SW | Cloud. | Sho'ry | Sho'ry |
| 12 | | ‘03 | 52 | 59 | 46 | 29·73 | 29·86 | | 54 | 52 | SSE | NE | Rain | Cloud. | Fine |
| 13 | | ‘63 | 56 | 60 | 48 | 29·90 | 29·91 | | 53 | 53 | NNW | ESE | Fine | Hail&Sto. | Rain |
| 14 | | | 52 | 61 | 48 | 30·06 | 30·02 | | 55 | 54 | NE | WNW | Cloud. | | |
| 15 | | | 52 | 59 | 49 | 29·84 | 29·92 | | 55 | 54 | WNW | NE | Cloud. | Sho'ry | Cloud. |
| 16 | | | 55 | 62 | 54 | 29·96 | 29·93 | | 54 | 54 | NW | N | Cloud. | | |
| 17 | | | 59 | 64 | 54 | 29·96 | 30·00 | | 55 | 55 | NW | WNW | Cloud. | | Sho'ry |
| 18 | ☽ | ‘54 | 60 | 64 | 54 | 30·06 | 29·91 | | 54 | 53 | WNW | SW | Fine | Fine | Cloud. |
| 19 | | ‘09 | 58 | 66 | 44 | 29·73 | 29·73 | | 57 | 51 | NW | W | Cloud. | | |

The quantity of rain fallen in May, is 2 inches and 63-100ths.

